# Public health approaches to ‘Leave No One Behind’ in heatwave resilience: insights from the UK

**DOI:** 10.1093/eurpub/ckae187

**Published:** 2024-11-21

**Authors:** Ana Raquel Nunes

**Affiliations:** Warwick Medical School, University of Warwick, Coventry, United Kingdom

## Abstract

Heatwaves pose significant threats to vulnerable populations, making resilience efforts crucial. This study aims to explore stakeholders' perspectives on heatwave resilience from a public health perspective, with a specific focus on operationalising the commitment to ‘Leave No One Behind’ (LNOB) as outlined in the United Nations 2030 Agenda for Sustainable Development. In-depth qualitative interviews were conducted with key stakeholders from national and local government, industry and business, academia, and civil society organizations. Interviews examined stakeholders' understanding of the progress and challenges associated with fulfilling the commitment of LNOB in the context of heat resilience from a public health perspective, in England, UK. Content analysis of interview transcripts was undertaken. Stakeholders emphasize the importance of equity, inclusivity, and public health priorities in heatwave resilience efforts while specifically addressing the commitment to LNOB. Disparities in vulnerability due to socioeconomic factors, challenges in identifying and supporting vulnerable populations, progress made in addressing heatwave resilience, and the role of government and society in improving resilience efforts were emphasized. Stakeholders highlighted the need for targeted interventions, strengthened community support networks, and policy changes to address systemic inequalities and promote inclusivity in resilience strategies. Stakeholders’ perspectives underscore the importance of aligning heatwave resilience efforts with global goals, particularly in promoting public health equity and inclusivity. By addressing the challenges identified and implementing the recommendations for improvement, policymakers and practitioners should work towards more equitable and inclusive resilience strategies to safeguard public health during heatwaves.

## Introduction

Heatwaves, exacerbated by climate change, are characterized by prolonged periods of excessively high temperatures that are increasingly frequent and intense [1], posing a significant threat to public health [2–6]. Heatwaves are associated with heat-related illnesses such as heatstroke, dehydration, and the exacerbation of pre-existing conditions [3–5, 7, 8]. Vulnerable populations, including older adults, children, individuals with pre-existing health conditions, those residing in substandard housing, the poor, and indigenous communities, suffer disproportionate impacts during and after heatwaves [9–11]. Socioeconomic factors exacerbate vulnerability, with disparities in income, access to assets, and societal attitudes contributing to uneven health outcomes, including morbidity and mortality [12, 13]. Additionally, heatwaves can impact infrastructure and exacerbate existing social, economic, and environmental inequalities.

As the frequency and severity of heatwaves accelerate, there is an urgent need to enhance resilience, particularly from a public health perspective [14, 15]. Efforts to enhance resilience to heatwaves increasingly emphasize inclusivity and equity [16, 17]. The UN’s 2030 Agenda for Sustainable Development, comprising 17 SDGs, addresses interconnected challenges such as climate change, health, and inequality [18]. Integrating climate action and resilience-building measures across various sectors can contribute to achieving multiple SDGs simultaneously, although with careful consideration of synergies and trade-offs [19]. Urgent action is imperative to achieve the SDGs by 2030 [20, 21]. However, there is a lack of clarity regarding countries’ strategies and policies to enhance heatwave resilience, with limited attention given to the relationship between achieving the SDGs and enhancing heatwave resilience [22].

Goal 3 of the SDGs is dedicated to ensuring healthy lives and promoting well-being for individuals of all ages [18], which is central to public health. It underscores the significance of health and well-being throughout every stage of life and advocates for universal health coverage and access to safe, effective, quality, and affordable medicines [21]. The commitment to ‘Leave No One Behind’ (LNOB), as outlined in the United Nations 2030 Agenda for Sustainable Development, emphasizes prioritizing vulnerable populations in resilience strategies [23]. However, achieving inclusive heatwave resilience requires a comprehensive understanding of the unique vulnerabilities and needs of different demographic groups [1, 24].

It is essential to prioritize climate-related concerns when developing and adapting public health strategies and policies [25], supporting populations at higher risk and most impacted by heatwaves that result in significant morbidity and mortality [3, 5]. The United Nations approach to LNOB seeks to reduce discrimination and rising inequalities within and among countries, striving to ensure progress for all individuals and population groups [23]. Improving heatwave resilience aligns with the sustainable development goals and is crucial for minimizing adverse impacts, particularly on public health [22]. The urgency of addressing heatwave resilience within the framework of the 2030 Sustainable Development Agenda prompts a critical examination. The LNOB framework’s focus on inclusivity and equity can be effectively applied to public health strategies for heatwave resilience. This ensures no vulnerable population is overlooked, promoting equitable health outcomes during extreme heat events.

This study critically examines the relevance of the LNOB framework in the context of heatwaves in England, using the UK as a case study. The research adopts a stakeholder-driven approach exploring perspectives on heatwave resilience from a public health standpoint, emphasizing the operationalization of LNOB. The study seeks to inform evidence-based policies and practices that safeguard public health during heatwaves while ensuring inclusivity and equity. The primary objective is to explore stakeholders’ perspectives on achieving the LNOB commitment, thereby informing policy, practice, and future research within the framework of the 2030 Sustainable Development Agenda. The research questions guiding this study include:

1. How do stakeholders perceive the commitment to LNOB in the context of heatwave resilience?
2. What are the current challenges and progress in operationalizing LNOB in heatwave resilience?
3. What specific interventions are recommended by stakeholders to enhance inclusivity and equity in heatwave resilience?

## Methods

A qualitative research design [26–28] was used to deliver the study’s aim through in-depth interviews [29, 30] with a diverse group of key stakeholders in England, UK. The interview guide was informed by the study’s aim and objectives, as well as relevant literature.

### Participant recruitment

Participant recruitment followed a purposive sampling technique to achieve maximum variability, complemented by snowball sampling to contact additional stakeholders. Four groups of key stakeholders were interviewed: (1) government, (2) private sector, (3) civil society, and (4) academia. Stakeholders were spread throughout the country covering both urban and rural areas. They were selected based on their experience, knowledge, and role in heatwave resilience policy and practice. The recruitment process began by contacting potential participants via email, inviting them to take part in an in-depth interview as part of the study. An information sheet detailing the study’s purpose, procedures, and ethical considerations was provided. Those who agreed to participate were then sent a consent form, which they were required to sign before the interview commenced.

### Ethical considerations

The research received ethical approval from the University of Warwick Research Ethics Committee. Participants were assured of their anonymity and confidentiality. Personal identifiers were removed from transcripts, and data was stored securely. Participants were informed that their involvement was voluntary, and that they could withdraw at any time without any repercussions.

### Selection of interviewees

The selection of interviewees from various disciplines is justified by the need for diverse perspectives to understand and address heatwave resilience comprehensively. Government officials provided insights into policy frameworks and regulatory measures; private sector representatives contributed knowledge on business continuity and infrastructure adaptation; civil society participants offered perspectives on community impacts and grassroots initiatives; and academics provided expertise on research findings and theoretical frameworks. This multidisciplinary approach ensured a holistic understanding of heatwave resilience.

### Data collection and analysis

Twenty-six interviews were conducted, audio-recorded, and transcribed verbatim. Each interview lasted between 20 and 60 min. Transcripts were exported to NVivo12^®^ software (QSR) for organizing and integrating data. Data were systematically coded to identify key issues and emergent patterns [31]. Content analysis was used to identify categories and themes, and commonalities within the qualitative data [32–35]. Text segments were categorized based on these categories and themes, with the aim of identifying areas of consensus and discrepancy. Through an iterative process, categories and themes were identified and refined using a combination of inductive and deductive coding, where initial categories and themes were established drawing from relevant literature.

### Quantitative content analysis

Additionally, quantitative content analysis was used to assess the frequency of the categories and themes identified. While the frequency of categories and themes alone may not necessarily indicate significance, it was calculated to facilitate the exploration of underrepresented or latent categories and themes and improve understanding of their prevalence.

## Results

A total of 26 key stakeholders were recruited, reflecting a diverse range of perspectives from various sectors including government, private sector, academia, and civil society. These stakeholders represented different disciplines such as health and social care, social science, technology, and science, as outlined in Tables 1 and 2. Table 1 illustrates that nine stakeholders were affiliated with national and local government, eight were from academia, six were from civil society, and three were from the private sector. Moreover, Table 2 highlights the representation of stakeholders across four main disciplinary areas. The health and social care sector comprised nine stakeholders, while the social science sector had eight stakeholders. Technology was represented by two stakeholders, and science by seven stakeholders. Notably, technology was the least represented discipline, with only two stakeholders.

**Table 1.** Stakeholders’ sectors

Sector	Description	Number
Government	National non-ministerial departments and executive agencies	4
	Local councils	5
Private	Business and industry	3
Civil society	Civil society organization and non-governmental organizations	6
Academia	Researchers and academics	8

**Table 2.** Stakeholders’ disciplinary background

Disciplinary background	Description	Number
Health and Social Care	Public health, social work	9
Social Science	Economics, geography, sociology, social policy, politics, and international studies	8
Technology	Environment and engineering	2
Science	Earth and environmental sciences, life sciences, sustainability	7

**Figure 1.**
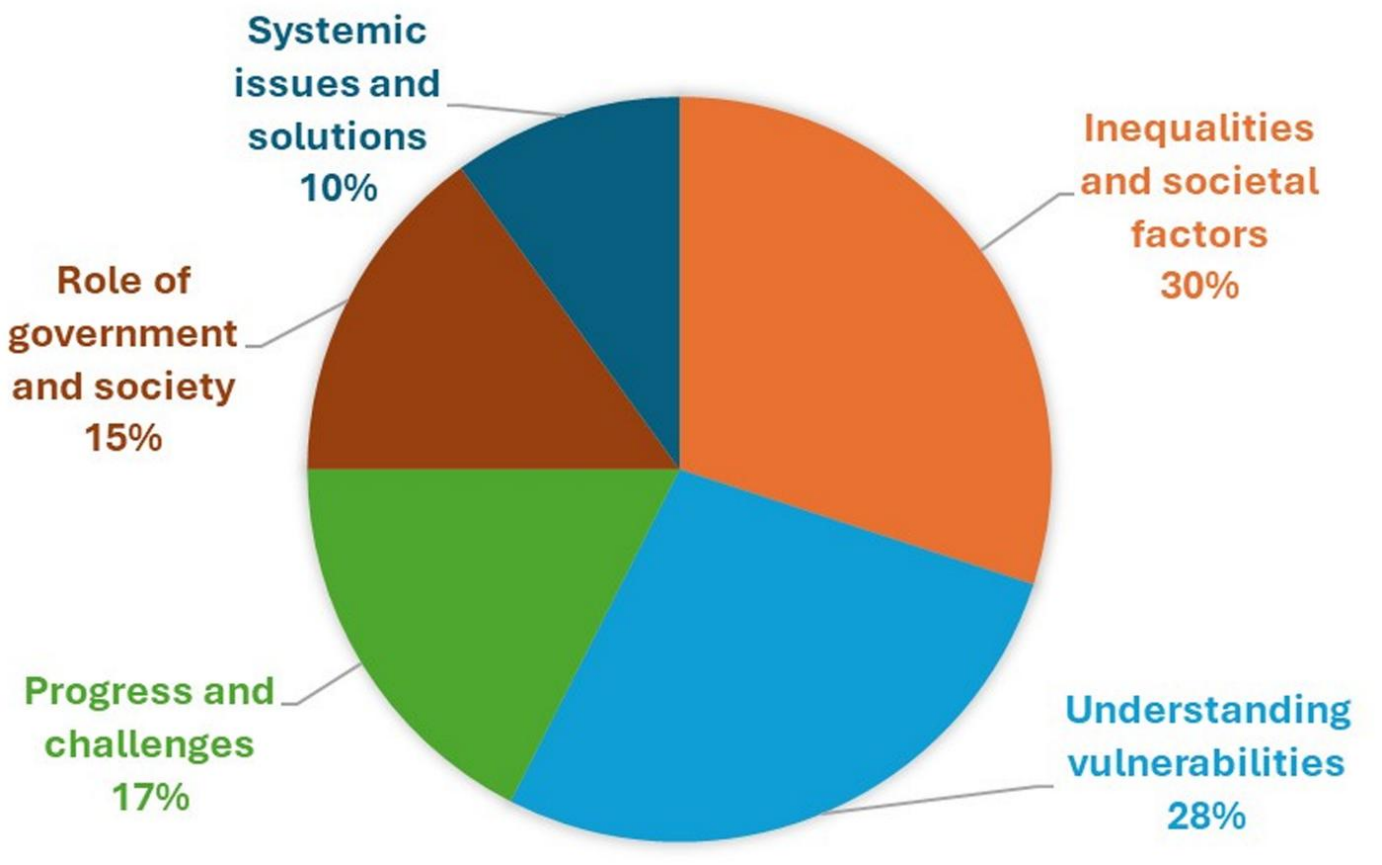
Frequency of themes associated to stakeholders’ understanding of ‘Leave No One Behind’ and how the pledge can be achieved for heatwave resilience.

Content analysis revealed several insights and important categories and themes associated with stakeholders’ understanding of LNOB in the context of heatwave resilience. To distil these, we undertook the frequency of commonly occurring categories and themes. The study also aimed to investigate how the pledge to LNOB can be achieved for heatwave resilience. The following five themes we identified from the analysis (also see Fig. 1): inequalities and societal factors (30%); understanding vulnerabilities (27.5%); progress and challenges (17.5%); role of government and society (15%); and systemic issues and solutions (10%). These percentages are approximate and subjective assessments based on the prominence and relevance of each theme within the interview transcripts. Below is presented a broad overview of the themes, along with illustrative quotes in Supplementary Material S1. It is crucial to note that certain topics and findings might overlap; therefore, these categories should not be viewed as fixed groupings.

### Inequalities and societal factors

Within this theme, stakeholders consistently highlight disparities in income, resource access, and societal attitudes as significant factors exacerbating vulnerability during heatwaves, profoundly impacting public health. They underscore the widening gap between wealthy and marginalized groups, driven by economic policies and societal attitudes that prioritize individual wealth over collective well-being. This recurring theme emphasizes the profound influence of socioeconomic disparities, access to resources/assets, and societal marginalization on vulnerability, particularly emphasizing how certain groups face disproportionate health risks during heatwaves due to systemic inequalities. However, perspectives on the extent and specific nature of these disparities vary across stakeholder groups. For instance, government officials and private sector representatives may acknowledge these inequalities but often highlight existing measures and policies aimed at mitigating these risks. In contrast, civil society and academic stakeholders tend to focus more on the inadequacy of current measures and the need for more radical changes to address these systemic issues effectively.

### Understanding vulnerabilities

Stakeholders stress the significance of identifying and supporting vulnerable populations from a public health perspective during heatwaves, including older adults, individuals with underlying health conditions, and those living in poor-quality housing. They emphasize the need to recognize the distinct vulnerabilities of various communities and demographics, such as minorities and urban dwellers affected by the urban heat island effect, who face increased health risks amid heatwaves but often lack adequate policy attention and intervention. This recurrent theme highlights the necessity of addressing diverse vulnerable groups while also acknowledging the challenges in effectively identifying and reaching them from a public health perspective. Health experts, for instance, often emphasize the need for more granular data to understand the specific health impacts on different vulnerable groups, whereas policymakers might focus more on broader intervention strategies. This difference in focus underscores the need for a more integrated approach that combines detailed health data with effective policy implementation.

### Progress and challenges

This theme encapsulates stakeholders’ perspectives on the progress made in increasing heatwave resilience from a public health perspective and the persisting challenges. While some express optimism regarding advancements, particularly in policy responsiveness and communication, many acknowledge the imperative for further action. Challenges include the need for sustained efforts, better communication strategies, and tackling broader societal issues such as poverty, inequality, and short-term political agendas, all acknowledged as barriers to progress. Stakeholders discuss both progress and challenges in addressing vulnerabilities, particularly in terms of health disparities and heatwave preparedness, emphasizing initiatives targeting vulnerable groups while recognizing the complexities in reaching them effectively from a public health perspective. Differences in perspective are evident here as well: Government and private sector stakeholders focus on the progress made and the effectiveness of current policies, while civil society and academic stakeholders highlight ongoing gaps and the need for more comprehensive, long-term strategies.

### Role of government and society

This theme focuses on stakeholders’ calls for stronger government action and societal support to improve heatwave resilience with a focus on public health considerations. They emphasize the need for better identification of vulnerable groups and individuals, investment in infrastructure, and policies aimed at mitigating health inequalities. Emphasis is placed on society’s role in supporting vulnerable populations through community networks, education, and reshaping societal attitudes toward taxation and wealth redistribution to promote public health equity. Stakeholders discuss the responsibilities of various stakeholders, including government, NGOs, charities, and community organizations, in addressing vulnerabilities, along with challenges such as bureaucratic obstacles and the need for effective networks to support vulnerable populations. While government officials highlight ongoing efforts and plans, civil society groups stress the need for more community-led initiatives and grassroots involvement to ensure that policies are effectively reaching those in need.

### Systemic issues and solutions

Stakeholders acknowledge systemic issues such as inadequate funding, short-term political agendas, and societal biases towards vulnerable populations as barriers to achieving heatwave resilience and public health equity. They propose solutions prioritizing equity, community engagement and involvement, and long-term sustainability, stressing the need for comprehensive strategies to effectively address these challenges. Proposed solutions include reshaping societal values towards inclusivity, improving access to resources/assets and support networks, and adopting a collective approach to heatwave resilience and public health equity. Stakeholders highlight systemic issues contributing to health vulnerabilities, such as inadequate housing, healthcare access, and social isolation, while discussing potential solutions involving better inter-organizational coordination and targeted public health interventions for at-risk groups. The emphasis on systemic solutions varies among stakeholders, with some focusing on policy reforms and others advocating for community-based approaches and stronger intersectoral collaboration.

### Consistent and inconsistent perspectives across stakeholder groups

Consistent themes include the acknowledgement of socioeconomic disparities, the need to identify and support vulnerable populations, and the recognition of systemic barriers to achieving heatwave resilience. However, inconsistencies emerge in the emphasis placed on specific issues and proposed solutions. Health experts stress the importance of detailed health data and targeted interventions, while government officials focus more on policy frameworks and existing measures. Private sector representatives highlight the need for business continuity and infrastructure adaptation, whereas civil society stakeholders emphasize community impacts and grassroots initiatives. These variations underscore the importance of a multidisciplinary approach that integrates diverse perspectives to develop comprehensive, effective strategies for enhancing heatwave resilience and public health equity.

### Operationalising LNOB

**Figure 2.**
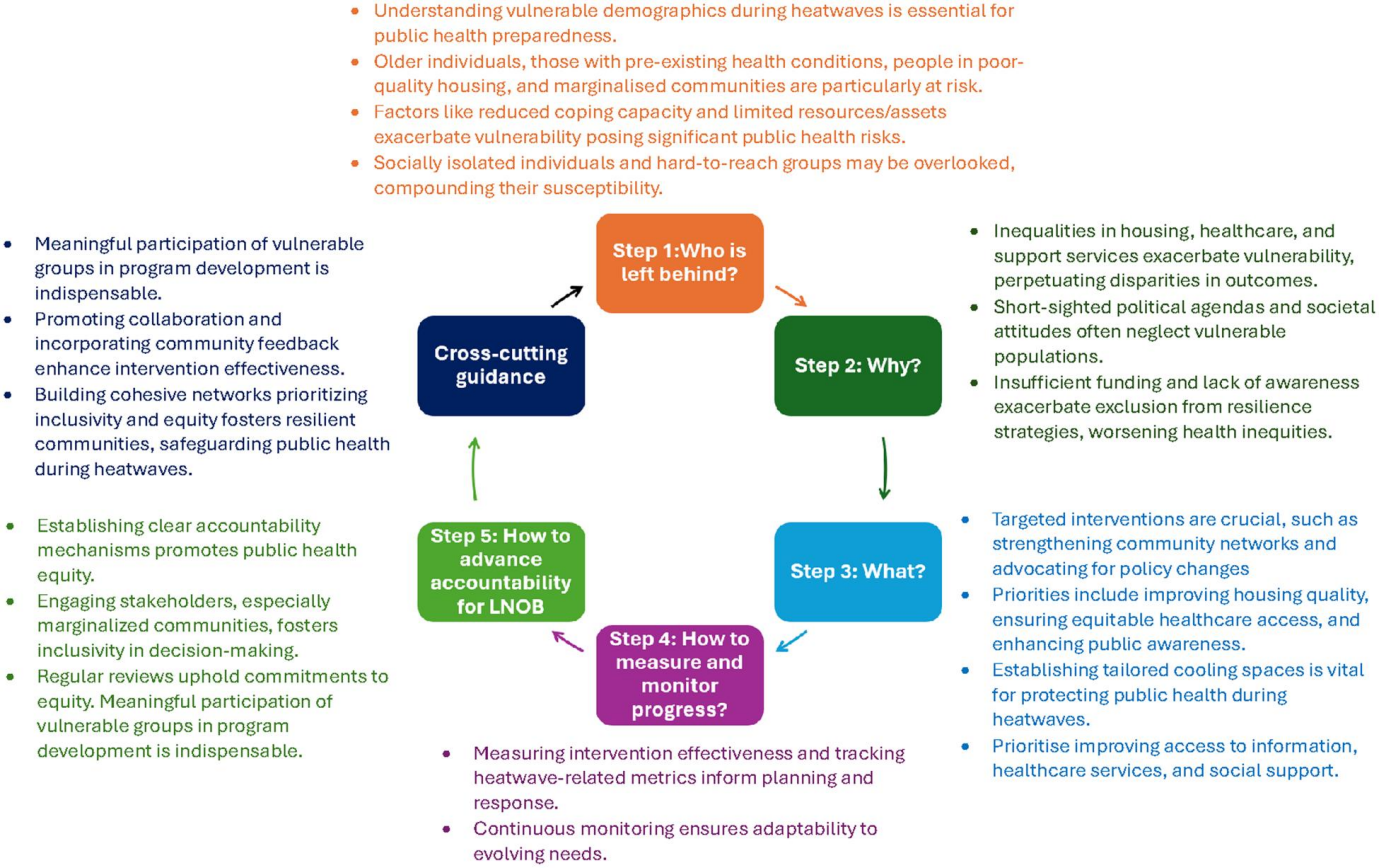
Operationalizing ‘Leave No One Behind’ in heatwave resilience.

Drawing on the interview data and operationalizing the commitment to LNOB as outlined by the United Nations Sustainable Development Group [23], a plan can be formulated as follows (also see Fig. 2).

#### Step 1: Who is left behind? Gathering the evidence

According to stakeholders’ interviews, understanding the demographics most at risk during heatwaves is crucial for building resilience while ensuring inclusivity. Based on the interview data, vulnerable populations include older individuals, those with health conditions, people in poor-quality housing, and marginalized communities. Stakeholders emphasize that these groups face increased vulnerability due to various factors such as reduced capacity to cope with extreme temperatures, barriers to accessing adequate housing, and lack of resources to cope with heat-related challenges. Additionally, socially isolated individuals and hard-to-reach groups with limited access to services may remain overlooked in heatwave planning and response efforts.

#### Step 2: Why? Prioritization and analysis

According to stakeholders in this study, the reasons for leaving people behind during heatwaves include inequalities in housing quality, healthcare access, and support services that exacerbate the risks faced by marginalized communities. Short-sighted political agendas often neglect the needs of vulnerable populations, while societal attitudes emphasizing individualism over collective support further perpetuate marginalization. Insufficient funding for public services compounds the issue, leaving vulnerable groups ill-equipped to cope with heat-related challenges. Additionally, a lack of awareness and understanding regarding the impact of heatwaves on vulnerable populations is highlighted as exacerbating their exclusion from resilience strategies.

#### Step 3: What? What should be done?

Actions to address the issue of LNOB during heatwaves mentioned by stakeholders include implementing targeted interventions, strengthening community support networks, and promoting policy implementation. These actions are aimed at improving housing quality, ensuring access to healthcare, and promoting equity and resilience across all demographics. Intensifying public awareness campaigns and establishing cooling spaces accessible to vulnerable groups are found to be vital components of these efforts. Furthermore, integrating equity considerations into urban planning and resilience initiatives is thought to be essential to foster inclusivity and address the unique needs of all communities. Strategies should thus, according to stakeholders, prioritize improving access to information, healthcare services, and social support for vulnerable populations during heatwaves, with a particular focus on targeted outreach efforts.

#### Step 4: How to measure and monitor progress

Measuring and monitoring progress are found essential for assessing the effectiveness of resilience efforts and ensuring inclusivity. Additionally, tracking heatwave-related mortality and morbidity among vulnerable groups provides crucial insights into the impact of mitigation efforts. Monitoring the accessibility and utilization of support services enables the identification of gaps and informs targeted interventions. Furthermore, surveys and assessments play a vital role in gauging public awareness and preparedness, guiding education, and outreach initiatives.

#### Step 5: How to advance accountability for LNOB

Establishing clear accountability mechanisms, engaging stakeholders, and advocating for policies that prioritize equity and inclusivity are crucial steps identified by stakeholders in advancing accountability for LNOB in heatwave resilience efforts. Additionally, active participation of all stakeholders, particularly those from marginalized communities, is found to be essential in decision-making processes to ensure their voices are heard and their perspectives considered.

#### Cross-cutting guidance: meaningful participation

Meaningful participation of vulnerable groups in decision-making processes and program development is highlighted by stakeholders as imperative to inclusive and effective resilience strategies. According to them, promoting collaboration among diverse stakeholders and incorporating community feedback and local knowledge increases the relevance and effectiveness of interventions. Engaging with a broad spectrum of stakeholders, including government agencies, charities, healthcare providers, and community organizations, is also essential to building cohesive networks and fostering collaborative efforts that prioritize inclusivity and equity in heatwave resilience actions.

### Integrating LNOB in planning and programming processes

To effectively improve heatwave resilience, it is imperative to comprehensively integrate LNOB principles throughout heatwave planning and programming processes. According to stakeholders, this involves adopting an inclusive approach that meticulously considers the diverse needs and capabilities of all population groups. Equity considerations must be intricately woven into every aspect of risk assessments, resource allocation, and policy development to ensure fair and just outcomes for all. Moreover, embedding the principles of LNOB into existing planning and programming processes, such as emergency response strategies and resilience-building initiatives, is crucial for fostering sustainable and inclusive heatwave resilience measures.

### Integrating LNOB in the context of improving heatwave resilience

In the pursuit of enhancing heatwave resilience, it is crucial to integrate LNOB principles, recognizing the disproportionate impact of heatwaves on vulnerable populations and prioritizing their needs in resilience efforts. Stakeholders acknowledge that this involves implementing targeted interventions that address the root causes of vulnerability, including housing inequality, healthcare access, and social isolation. Collaboration across sectors is considered essential to develop comprehensive strategies for reducing heatwave risks and safeguarding all members of society. Moreover, stakeholders highlighted that specific strategies must be developed to address the unique vulnerabilities of different populations during heatwaves, such as the elderly, socially isolated individuals, and those living in poor housing conditions.

### Pathways to LNOB

Stakeholders’ quotes in Supplementary Material S2 showcase how the pledge to LNOB can be achieved for heatwave resilience. Stakeholders’ accounts underscore the importance of identifying and supporting vulnerable populations, tailoring interventions to meet their specific public health needs to ensure inclusivity. Recognizing barriers such as language obstacles and social isolation can inform strategies to improve outreach and communication, vital for reaching vulnerable populations effectively from a public health standpoint. Emphasizing intersectoral collaboration and partnerships between government agencies, healthcare providers, and community organizations increases response efforts and support for vulnerable groups is considered key. Stakeholders acknowledged limited progress and policy gaps, underscoring the need for continuous evaluation and improvement to effectively safeguard public health for all individuals and communities.

## Discussion

This study’s findings closely align with existing literature on heatwave resilience and inclusivity [2, 4, 36]. Numerous studies have highlighted the disproportionate impact on marginalized communities, citing income inequality, lack of resources, and societal attitudes as exacerbating vulnerability [2, 5, 6, 10]. The recognition of understanding vulnerabilities and supporting vulnerable populations aligns with research identifying at-risk groups like the elderly, those with health conditions, and people in poor-quality housing during heatwaves [12, 13]. The progress in heatwave resilience, supported by stakeholder perspectives, mirrors recent developments in the UK [37]. Stakeholders acknowledged efforts in infrastructure, policy, and community awareness. Notably, the UK’s Health Security Agency (UKHSA) adverse weather and health plan reflects the country’s proactive approach to safeguarding health during heatwaves, showcasing the progress made [37]. Stakeholders underscored the critical role of government and society in implementing heatwave resilience strategies, echoing calls for collaboration across sectors. The emphasis on public health priorities resonates with global and national policies emphasizing health equity and inclusivity, such as the UN’s 2030 Agenda and the UKHSA’s initiatives. Targeted interventions and community support networks, as highlighted by stakeholders, align with evidence-based recommendations for enhancing resilience. Stakeholders identified systemic inequalities exacerbated by heatwaves, underscoring the need for an inclusive approach to resilience. This study’s insights align with global goals, particularly in promoting public health equity and inclusivity in heatwave resilience efforts.

Acknowledging challenges in reaching vulnerable groups and calls for stronger government action resonate with literature documenting barriers to accessing heatwave resilience among vulnerable populations [16, 17]. Identified systemic issues, such as inadequate funding and short-term political agendas, align with scholarly discourse emphasizing comprehensive strategies for inclusivity in heatwave resilience efforts [38, 39]. The steps for operationalizing LNOB in heatwave resilience efforts reflect recommendations from academic literature, emphasizing evidence-based interventions, prioritizing vulnerable populations, targeted strategies, and robust monitoring and evaluation [1–3].

## Conclusions

Stakeholders’ perspectives reveal the complexity of achieving heatwave resilience while operationalizing LNOB in England. The findings highlight the importance of equity, inclusivity, and public health priorities in resilience efforts. Addressing socioeconomic disparities, supporting vulnerable populations, and leveraging progress in policy and infrastructure are essential. The commitment to LNOB, as outlined in the UN’s 2030 Agenda, provides a critical framework for guiding resilience strategies.

## Supplementary Material

ckae187_Supplementary_Data

## Data Availability

The data underlying this article cannot be shared publicly due to privacy of individuals who participated in the study. Key points Supporting vulnerable groups such as the elderly, those with health conditions, and people in poor-quality housing is critical during heatwaves. Progress in the UK on heatwave resilience is seen through infrastructure, policy, and community awareness. Effective heatwave resilience strategies require collaboration across government and society, aligning with global and national policies on health equity and inclusivity. Implementing the LNOB framework in heatwave resilience involves prioritizing vulnerable populations, evidence-based interventions, and robust monitoring, while addressing systemic inequalities and fostering cross-sector collaboration.
