# Supplementary material for: Public health approaches to ‘Leave No One Behind’ in heatwave resilience: insights from the UK
Source: Eur J Public Health. 2024 Nov 21;35(1):171–7. doi: 10.1093/eurpub/ckae187 (PMC11832139; doi:10.1093/eurpub/ckae187)
Supplement: ckae187_Supplementary_Data [file ckae187_supplementary_data.zip › ckae187_Supplementary_Data/ejph-2024-07-om-0479-File006.docx]

**Supplementary Material 1.** **Illustrative quotes according to themes**

| Inequalities and societal factors | "White, middle class, typically male, retired in age want to do the right thing but might not be thinking about everyone in their community because they are all from a particular demographic themselves." (S8) |
| --- | --- |
|  | "Isolated older people living alone at home are a high risk group; older people in poor housing [...]" (S10) |
|  | "[…] it’s horrendous what is happening. And the problem is that climate change will only increase these inequalities." (S17) |
|  | "[…] there are a lot of vulnerable people, such as those with learning difficulties, old folk, lonely folk, those that can’t get out and look after themselves [...] I don’t think they are being brought along." (S23) |
| Understanding vulnerabilities | "Struggle to get commitment from anybody. That has been the major problem for resilience everywhere; How you reach the most vulnerable people in the community, socially isolated, isolated geographically?" (S4) |
|  | "The poor, the old, the very young probably, who are misfortunately affected by all sorts of environmental insults, including disease, air pollution, etc., etc." (S9) |
|  | "[…] it’s about assessing who is the most vulnerable, who is the most at risk from heatwaves and ensuring that they aren’t forgotten." (S16) |
|  | "[…] for most people most healthy people, they just need to follow very simple measures about keeping out of strong sunlight, keeping themselves hydrated and cool." (S21) |
| Progress and challenges | "[…] it’s about health inequalities, poor people, on low income, are generally more affected by things like heatwaves. Therefore, they are more left behind." (S5) |
|  | "We have only now reached the awareness that it needs to be done." (S10)  "We are getting better at it. There is much more sensitive policy, communication and media approach regarding the people who might be suffering disproportionately from acute heatwaves." (S14) |
|  | "We are making some progress but it’s going to be a long journey." (S22) |
| Role of government and society | "Recognising that the most vulnerable members of society are most at risk, that includes people at either end of life, the very old, the very young; but also very much the very poor [...]" (S7) |
|  | "The national plan does go towards that to a degree, to provide various levels of information for care home professionals but not ordinary members of the public." (S12) |
|  | "If we were improving inequalities and life expectancy was increasing than I suppose the argument would be to do more of that, make sure we do it in a way we have an eye on climate change and future impacts and not just focusing on the current impacts." (S17) |
|  | "[…] we need sustained action on a consistent basis to ensure we’re not leaving everyone behind." (S16) |
| Systemic issues and solutions | "[…] there needs to be work done on whether the most vulnerable people are being reached or not." (S13) |
|  | "Certainly GPS as one, there are lots of organisations like ‘Meals on Wheels’ who have contact with vulnerable individuals, charities like ‘Age UK’ they know people who are most vulnerable." (S13) |
|  | "[…] our political economy is based on competition and valuing individuals in terms of their market value [...] Our system is based on leaving people behind." (S19) |
|  | "It’s a challenge to do that. It’s probably other examples from outside of this sector of sustainable development or improving resilience training or embedding learning from other sectors on how to ensure that particular individuals are not left out." (S20) |
