# Supplementary material for: Public health approaches to ‘Leave No One Behind’ in heatwave resilience: insights from the UK
Source: Eur J Public Health. 2024 Nov 21;35(1):171–7. doi: 10.1093/eurpub/ckae187 (PMC11832139; doi:10.1093/eurpub/ckae187)
Supplement: ckae187_Supplementary_Data [file ckae187_supplementary_data.zip › ckae187_Supplementary_Data/ejph-2024-07-om-0479-File007.docx]

**Supplementary Material 2.** **Illustrative quotes showcasing how the pledge to ‘Leave No One Behind’ can be achieved for heatwave resilience**

| "Leaving no one behind. This strapline has been used in many different contexts [...] It’s part of our core fabric, looking at who is most vulnerable. It’s what we do anyway." (S2) |
| --- |
| "It’s all about identifying those that are most vulnerable [...] Make sure we work together to achieve health." (S3) |
| "It’s about health inequalities, poor people, on low income, are generally more affected by things like heatwaves [...] But it has to be targeted to the right people for it to work properly." (S5) |
| "Recognising that the most vulnerable members of society are most at risk [...] Those being left behind are the homeless, people who can’t afford good accommodation [...] It’s always usually the poor who suffer the most." (S7) |
| "Everyone in the community needs to be considered not just a selected few [...] Needs to be more inclusive." (S8) |
| "With the exception perhaps of the old, it’s because the other two demographics are not catered for in most political decisions [...] Improving the environment of our cities [...] Reducing the burden of environmental insults on people." (S9) |
| "Making sure that everyone has access to the same information [...] The challenge is to make sure that they are informed of the risks." (S12) |
| "Some warning systems may require GPs to contact the most vulnerable people [...] So some kind of measures like that I think have the potential to make sure that nobody is left behind." (S13) |
| "It’s making sure that we look across generations and create solutions that the old and frail aren’t neglected [...] We shouldn’t leave anyone behind." (S14) |
| "It’s about assessing who is the most vulnerable, who is the most at risk from heatwaves and ensuring that they aren’t forgotten [...] It’s ensuring that all of those that represent different groups within society [...] have a voice in the decision-making processes." (S16) |
| "I think unfortunately we are already leaving people behind [...] We need to do as much as we can to reduce those inequalities anyway so that those populations are more resilient." (S17) |
| "We know that those who are most at risk during heatwaves are usually people who are vulnerable because of their health [...] And there are usually people on lower incomes and who are cut off perhaps from other forms of community support and we need to strengthen that community support." (S21) |
| "I would say that is an incredibly ambitious thing to think about given the amount of people we leave behind already in our current systems [...] It would be great, but we are so far behind the curb [...] Who is being left behind? There is a long list, but I think there are a lot of vulnerable people [...]" (S23) |
